# Supplementary material for: Epigenetic Regulation and Molecular Mechanisms of Burn Injury-Induced Nociception in the Spinal Cord of Mice
Source: Int J Mol Sci. 2024 Aug 4;25(15):8510. doi: 10.3390/ijms25158510 (PMC11313498; doi:10.3390/ijms25158510)
Supplement: Supplementary file 1 [file ijms-25-08510-s001.zip › ijms-3142642-supplementary.pdf]

Supplementary Table S1.

Assessment of Histone H3 PTMs in the dorsal horn of spinal cord post-burn injury using dual immunolabeling
Quantification of histone H3 PTM levels in DAPI-labeled cells following burn injury, expressed as a percentage of the DAPI-labeled cells.

	**Ipsilateral** (mean ± SEM)	**Contralateral** (mean ± SEM)	**p-value**
**Total Histone H3**	61.4 % ± 10.5	49.8 % ± 9.3	0.39
**H3K4me1**	33.4 % ± 12.7	20.2 % ± 6.5	0.05
**H3K4me2**	36.4 % ± 2.3	27.8 % ± 2.4	0.045
**H3K4me3**	38.0 % ± 2.9	23.0 % ± 5.5	0.03
**H3K4me3K9ac**	35.5 % ± 8.8	29.2 % ± 15.9	0.279
**p-S10H3**	6.09 % ± 0.6	4.14 % ± 1.9	0.044

Supplementary Table S2.
Quantification of histone H3 PTMs colocalization with Pdyn neurons in DAPI-labeled cells post-burn injury, expressed as a percentage of the DAPI-labeled cells.

	**Ipsilateral** (mean ± SEM)	**Contralateral** (mean ± SEM)	**p-value**
**Total Histone H3**	2.62 % ± 0.4	1.64 % ± 0.15	0.26
**H3K4me1**	1.95 % ± 0.9	1.07 % ± 0.5	0.07
**H3K4me2**	1.85 % ± 0.8	1.65 % ± 0.4	0.5
**H3K4me3**	1.79 % ± 0.14	1.14 % ± 0.4	0.07
**H3K4me3K9ac**	2.2 % ± 0.4	1.59 % ± 0.9	0.19
**p-S10H3**	1.11 % ± 0	0.74 % ± 0.3	0.012

Supplementary Table S3.
Quantification of histone H3 PTMs colocalization with Pdyn Neurons post-burn injury, expressed as a percentage of Pdyn neurons.

	**Ipsilateral** (mean ± SEM)	**Contralateral** (mean ± SEM)	**p-value**
**Total Histone H3**	76.9 % ± 23.0	88.6 % ± 2.9	0.33

**H3K4me1**	62.7 % ± 7.2	37.5 % ± 12.5	0.11
**H3K4me2**	53.84 % ± 15.38	44.7 % ± 1.9	0.3
**H3K4me3**	74.7 % ± 19.9	63.7 % ± 16.9	0.34
**H3K4me3K9ac**	82.6 % ± 6.2	65.7 % ± 5.7	0.09
**p-S10H3**	59.0 % ± 3.4	31.8 % ± 4.5	0.02

Supplementary Table S4.

The list of the top 15 up and down-regulated differently expressed genes (DEGs) in the spinal cord in response to burn injury

	Gene symbol	Gene name	Alteration in gene expression	Molecular function	Biological process	Fold-change vs control
1	**RNASET2**	ribonuclease 6	↑	RNA endonuclease activity	immune system process	1.91
2	**COMP**	Cartilage Oligomeric Matrix Protein	↑	cell adhesion molecule	negative regulation of the apoptotic process	1.63
3	**FOSB**	FBJ osteosarcoma oncogene B	↑	DNA binding	Negative regulation of transcription	1.52
4	**ACP5**	Tartrate-Resistant Acid Phosphatase 5a	↑	acid phosphatase activity	negative regulation of nitric oxide biosynthetic process<br>negative regulation of macrophage cytokine production	1.52
5	**RASGRP4**	RAS Guanyl Releasing Protein 4	↑	guanyl-nucleotide exchange factor activity	MAPK signaling pathway<br>transmembrane receptor protein tyrosine kinase signaling pathway	1.5
6	**PRSS53**	Polyserine Protease 3	↑	serine-type peptidase activity	proteolysis	1.5
7	**GLT8D2**	Glycosyltransferase 8 Domain Containing 2	↑	UDP-glycosyltransferase activity	None by GO term	1.48
8	**ERG1**	early growth response 1	↑	Transcriptional regulator	Negative regulation of transcription	1.39
9	**MYH2**	Myosin Heavy Chain 2a	↑	microfilament motor activity	muscle contraction	1.35
10	**GNGT2**	G Protein Subunit Gamma Transducin 2	↑	guanine nucleotide-binding protein	G protein-coupled receptor signaling pathway	1.33

11	**BANK1**	B Cell Scaffold Protein With Ankyrin Repeats 1	↑	B-cell receptor-induced calcium mobilization	positive regulation of MAPK cascade negative regulation of interleukin-6 production	1.33
12	**TRPC6**	Transient Receptor Potential Cation Channel Subfamily C Member 6	↑	receptor-activated non-selective calcium permeant cation channel	BDNF-TrkB signaling and positive regulation of cytosolic calcium ion concentration	1.33
13	**GPR150**	G protein-coupled receptor 150	↑	Signaling receptor activity	GPCR downstream signaling	1.29
14	**SOCS2**	suppressor of cytokine signaling 2	↑	Negative regulation of cytokine signal transduction	activation of JAK/STAT transcription pathway	1.29
15	**CPT1B**	carnitine Palmitoyltransferase 1B	↑	carnitine O-palmitoyltransferase activity	fatty acid metabolic process	1.28
16	**VGLL3**	Vestigial Like Family Member 3	↓	Protein binding	regulation of transcription by RNA polymerase II	-1.21
17	**CCDC27**	Coiled-Coil Domain Containing 27	↓	Protein binding	None by GO term	-1.23
18	**FOXQ1**	Forkhead Box Q1	↓	DNA binding	negative regulation of transcription by RNA polymerase II	-1.23
19	**RBM15**	RNA Binding Motif Protein 15	↓	RNA-binding protein	regulation of alternative mRNA splicing and positive regulation of transcription	-1.26
20	**LMLN**	Leishmanolysin like peptidase	↓	zinc-metallopeptidase	proteolysis	-1.30
21	**D430019H16Rik**	RIKEN cDNA D430019H16 gene	↓	unknown	None by GO term	-1.31
22	**SH3D21**	SH3 Domain Containing 21	↓	Protein binding	Cell migration	-1.31

23	**UNC5D**	Unc-5 Netrin Receptor D	↓	Receptor for the netrin NTN4	axon guidance<br>cell-cell adhesion via plasma-membrane adhesion molecules	-1.34
24	**SPAG8**	Sperm Associated Antigen 8	↓	cytoskeletal protein binding	cell differentiation	-1.36
25	**SAMD5**	Sterile Alpha Motif Domain Containing 5	↓	protein binding	None by GO term	-1.41
26	**6430571L13Rik**	RIKEN cDNA 6430571L13 gene	↓	unknown	None by GO term	-1.42
27	**RXFP1**	Relaxin Family Peptide Receptor 1	↓	receptor for relaxins	G protein-coupled receptor signaling pathway	-1.47
28	**ADORA3**	Adenosine Receptor A3	↓	G-protein-coupled adenosine receptor	G protein-coupled adenosine receptor signaling pathway	-1.53
29	**MMP28**	Matrix Metalloproteinase-28	↓	proteolysis of extracellular matrix	tissue homeostasis and burn wound repair	-1.60

Supplementary Table S5.

The list of the top 15 up and down-regulated DEGs in the spinal cord in response to formalin application

	Gene symbol	Gene name	Alteration in gene expression	Molecular function	Biological process	Fold-change vs control
1	**MT-ATP8**	Mitochondrially Encoded ATP Synthase 8	↑	ATP hydrolysis activity and proton transmembrane transporter activity	ATP biosynthetic process	4.14
2	**MT-CO2**	Cytochrome C Oxidase Subunit 2	↑	Component of the cytochrome c oxidase	ATP synthesis coupled electron transport	2.43
3	**NCAPH**	Non-SMC Condensin I Complex Subunit H	↑	Regulatory subunit of the condensin complex	positive regulation of chromosome condensation	1.86
4	**4930539E08Rik**	BCL2 interacting protein 5	↑	Unknown	None by GO term	1.66
5	**TMEM181**	Transmembrane Protein 181	↑	G protein-coupled receptor	None by GO term	1.61
6	**CD93**	CD93 Molecule	↑	Receptor for C1q	macrophage activation<br>cell adhesion	1.52
7	**1190007I07Rik**	ubiquinol-cytochrome c reductase complex assembly factor 6	↑	required for the assembly and stability of the mitochondrial ubiquinol-cytochrome c reductase complex	mitochondrial respiratory chain complex III assembly	1.35
8	**DNAH3**	Dynein Axonemal Heavy Chain 3	↑	ATP hydrolysis activity and microtubule motor activity	microtubule-based movement	1.31
9	**SYNE4**	Spectrin Repeat Containing Nuclear Envelope Family Member 4	↑	actin binding	establishment of epithelial cell apical/basal polarity	1.25

10	**REL**	REL Proto-Oncogene, NF-KB Subunit	↑	DNA-binding transcription factor activity	negative regulation of gene expression	1.25
11	**Colca2**	POU class 2 homeobox associating factor 3	↑	Transcriptional coactivator	positive regulation of DNA-templated transcription	1.25
12	**IL1RAPL2**	Interleukin 1 Receptor Accessory Protein Like 2	↑	NAD+ nucleosidase activity	cytokine-mediated signaling pathway<br>regulation of presynapse assembly	1.21
13	**Ago4**	argonaute RISC catalytic subunit 4	↑	nucleic acid binding and double-stranded RNA binding	regulation of translation	1.16
14	**FANCD2**	Fanconi anemia, complementation group D2	↑	DNA polymerase binding	DNA repair and DNA damage response	1.15
15	**CCN1**	Cellular Communication Network Factor 1	↑	extracellular matrix binding	wound healing<br>matrix remodeling	1.15
16	**EIF4E**	eukaryotic translation initiation factor 4E	↓	RNA binding and translation initiation factor activity	translational initiation	-1.22
17	**IFIT1BL11**	interferon induced protein with tetratricpeptide repeats 1B like 1	↓	Interferon-induced antiviral RNA-binding protein	cellular response to type I interferon	-1.26
18	**LRRC3**	Leucine Rich Repeat Containing 3	↓	Protein binding	None by GO term	-1.28
19	**H4C11**	H4 Clustered Histone 11	↓	Core component of nucleosome	Transcription regulation, DNA repair, nucleosome remodeling	-1.29
20	**GM10320**	SEC61 Translocon Subunit Beta	↓	ribosome receptor	post-translational protein targeting to membrane, translocation	-1.31
21	**GADL1**	Glutamate Decarboxylase-Like Protein 1	↓	aspartate 1-decarboxylase activity	carboxylic acid metabolic process	-1.35

22	**DAPL1**	Death Associated Protein Like 1	↓	death domain binding activity	apoptotic signaling pathway	-1.40
23	**OMD**	Osteomodulin	↓	Protein binding	Cell adhesion	-1.42
24	**ZFP185**	Zinc Finger Protein 185 With LIM Domain	↓	Nucleic acid-binding protein	None by GO term	-1.44
25	**ADAT2**	Adenosine Deaminase TRNA Specific 2	↓	tRNA-specific adenosine deaminase activity	tRNA processing	-1.51
26	**LST1**	Leukocyte Specific Transcript 1	↓	membrane protein	dendrite development immune system process	-1.51
27	**CDCA7**	Cell Division Cycle Associated 7	↓	transcriptional regulator	Validated targets of c-myc transcriptional activation and apoptotic process	-1.53
28	**PROSER3**	Proline And Serine Rich 3	↓	Protein binding	None by GO term	-1.58
29	**C1QTNF1**	Complement C1q Tumor Necrosis Factor-Related Protein 1	↓	Protein binding activity	positive regulation of MAPK cascade	-1.68

Supplementary Table S6.

The list of the commonly up and down-regulated DEGs in the spinal cord in response to the two experimental pain models used (burn injury model and inflammatory pain model)

	Gene symbol	Gene name	Alteration in gene expression	Molecular function	Biological process	Fold-change *vs* control*
1	**HBA-A2**	Hemoglobin Subunit Alpha 2	↑	oxygen carrier activity	Cellular responses to stress	2.92 / 2.94
2	**MT-ATP6**	Mitochondrially Encoded ATP Synthase 6	↑	proton-transporting ATP synthase activity	ATP biosynthetic process	2.39 / 2.80
3	**EGR4**	Early Growth Response 4	↑	transcriptional regulator	positive regulation of transcription by RNA polymerase II	2.36 / 1.10
4	**DEGS2**	Delta 4-Desaturase, Sphingolipid 2	↑	sphingolipid delta-4 desaturase activity<br>sphingosine hydroxylase activity	lipid biosynthetic process	1.48 / 1.08
5	**CUBN**	Cubilin	↑	Endocytic receptor	lipid metabolic process	1.36 / 1.15
6	**PRICKLE3**	Prickle Planar Cell Polarity Protein 3	↑	Protein binding	mitochondrial membrane ATP synthase assembly<br>component of atypical Wnt-signaling	1.23 / 1.15
7	**SYCP2**	Synaptonemal Complex Protein 2	↑	DNA binding	negative regulation of apoptotic process	1.20 / 1.21
8	**RPS26-PS1**	Ribosomal Protein S26	↑	RNA binding	negative regulation of RNA splicing	1.16 / 1.65
9	**KCNA3**	Voltage-Gated Potassium Channel Protein Kv1.3	↑	potassium voltage-gated channel	potassium ion transmembrane transport	1.05 / 1.52

10	**BTG2**	BTG Anti-Proliferation Factor 2	↑	transcription corepressor activity	negative regulation of transcription by RNA polymerase II	1.04 / 1.08
11	**ADA**	Adenosine Deaminase	↑	adenosine deaminase activity	purine metabolism and in adenosine homeostasis	1.01 / 1.12
12	**GCH1**	GTP Cyclohydrolase 1	↑	calcium ion binding and GTP binding	tetrahydrobiopterin biosynthetic process<br>nitric oxide biosynthetic process	1.018 / 1.00
13	**ALOXE3**	Arachidonate Lipoxygenase 3	↑	hydroperoxide isomerase activity	Fatty acid and arachidonic acid metabolism	1.01 / 1.15
14	**KIF23**	Kinesin Family Member 23	↑	cytoskeletal motor activity	microtubule-based movement	1.01 / 1.15
15	**EXPH5**	Exophilin-5	↓	Rab effector protein	intracellular vesicle trafficking, positive regulation of exocytosis	-1.03 / -1.61
16	**HFE2**	Hemojuvelin BMP Co-Receptor	↓	protein binding	regulation of transcription by RNA polymerase II	-1.05 / -1.35
17	**CK137956**	cDNA sequence CK137956	↓	Unknown	None by GO term	-1.08 / -1.04
18	**MUSTN1**	Musculoskeletal, Embryonic Nuclear Protein 1	↓	unknown	positive regulation of gene expression<br>positive regulation of the proteoglycan biosynthetic process	-1.11 / -1.35
19	**CBX2**	chromobox 2	↓	Transcriptional repressor	negative regulation of transcription by RNA polymerase II	-1.18 / -1.26
20	**GM5901**	predicted gene 5901	↓	Unknown	None by GO term	-1.28 / -1.35
21	**CAR15**	carbonic anhydrase 15	↓	hydration of carbon dioxide	regulation of the apoptotic process	-1.82 / -1.25

* Fold change values in burn injury and formalin treatment, respectively.
